# Enhancing oxidative stability and sensory properties of ostrich meat using *Malva neglecta* mucilage nanocomposite coating loaded with *Myrtus communis* essential oil during refrigeration

**DOI:** 10.1016/j.fochx.2025.102455

**Published:** 2025-04-09

**Authors:** Atefeh Karimi, Majid Aminzare, Hassan Hassanzadazar, Mahsa Hashemi, Shahin Roohinejad, Alaa El-Din Ahmed Bekhit, Reza Tahergorabi

**Affiliations:** a Zanjan Pharmaceutical Biotechnology Research Center, Zanjan University of Medical Sciences, Zanjan 4513956184, Iran; b Department of Food Safety and Hygiene, School of Public Health, Zanjan University of Medical Sciences, Zanjan 4515786349, Iran; c Division of Food and Nutrition, Burn and Wound Healing Research Center, Shiraz University of Medical Sciences, Shiraz, Iran; d Department of Food Science, University of Otago, Dunedin 9016, New Zealand; e Food and Nutritional Sciences Program, North Carolina Agricultural and Technical State University, Greensboro, NC 27411, USA

**Keywords:** Nanocomposite, *Malva neglecta* mucilage, *Myrtus communis* essential oil, Oxidative stability, Sensory properties, Ostrich meat

## Abstract

Ostrich meat has a favorable nutritional profile but is susceptible to oxidative deterioration due to its high prooxidants. This study aimed to assess the effects of edible coatings made from *Malva neglecta* mucilage (MLM) including *Myrtus communis* essential oil (MEO), in both conventional and nanocomposite (nanoclay-based) forms on the oxidative stability and sensory characteristics of ostrich meat during 21-day storage at 4 °C. Samples coated with nanocomposite MLM containing 8 % MEO (NMLM-MEO 8 %) showed significantly lower pH (6.19), peroxide value (1.67 meq/kg lipid), thiobarbituric acid reactive substances index (1.11 mg MDA/kg), and carbonyl content (1.74 nmol/mg protein), alongside higher phenolic content (3.51 mg GAE/g meat) and overall acceptability score (4.6) compared to other groups (*P* ≤ 0.05). These findings demonstrate the potential of NMLM-MEO 8 % coating containing natural antioxidants as effective active packaging material, providing oxidative protection and sensory improvement in ostrich meat, while offering a sustainable alternative for the meat packaging industry.

## Highlights

• Conventional and nanocomposite MLM-MEO coatings were prepared and characterized.
• Ostrich meat was treated with MLM-MEO and NMLM-MEO coatings and stored at 4 °C.
• The oxidative and sensory stability of ostrich meat samples were compared.
• The NMLM-MEO 8 % coating demonstrated the highest antioxidant activity.
• Ostrich meat with NMLM-MEO 8 % showed better sensory attributes than other groups.

## 1 Introduction

Ostrich meat is recognized for its favorable fatty acid profile, characterized by a balanced proportion of polyunsaturated to saturated fatty acids (PUFA/SFA) and an optimal balance of omega-6 to omega-3 fatty acid (n-6/n-3). Furthermore, its low intramuscular fat and sodium content, coupled with elevated levels of iron and vitamin E, render it a significant component of the dietary intake (Poławska et al., 2011). However, the presence of prooxidants such as heme iron and PUFA make ostrich meat susceptible to oxidative deterioration. Oxidation can compromise the nutritional quality of the meat during storage, leading to undesirable flavors, spoilage, and the potential formation of harmful compounds detrimental to consumer health. Therefore, it is imperative to mitigate oxidation and the production of harmful byproducts in ostrich meat (Horbańczuk et al., 2021). The implementation of active packaging systems that incorporate antioxidant compounds presents an efficient strategy for inhibiting oxidative processes in meat (Amiri et al., 2019).

In light of increasing environmental concerns about the negative impacts of plastic packaging waste, a range of alternatives has been explored, including biodegradable edible polymers, for active food packaging systems. Plant-derived mucilages, which are natural polysaccharide polymers, are frequently used in the food sector for producing edible coatings (Heydari et al., 2020). *Malva neglecta*, commonly referred to as mallow, is an annual plant characterized by its purple blooms and lightly lobed leaves. In Iran, it is known as Panirak and has a long-standing tradition of use in the treatment of skin wounds, bronchitis, digestive issues, and inflammation. Several studies have examined the potential of mucilage sourced from different parts of *M. neglecta* for the development of edible coatings applicable to different types of meat (Abbasi et al., 2024). Polymer nanocomposites are mainly formed by incorporating nanofillers, such as nanoclay, into a polymer matrix to boost the mechanical performance of food packaging systems. Nanoclays are favored nanomaterials in food packaging applications and are extensively employed in biodegradable coatings due to their natural origin, affordability, biocompatibility, high thermal stability, and barrier properties (Nath et al., 2022). However, regulations have been established concerning the transfer of clay nanoparticles from packaging to food products. Aluminum and silicon ions were identified as indicators of this migration, which is influenced by nanoclay content, storage duration, and temperature (Paidari et al., 2021).

Increasing awareness of the adverse health effects associated with chemical preservatives has generated a significant interest in exploring natural alternatives for active packaging (Heydari et al., 2020). In this regard, active nanocomposites containing essential oils (EOs) have been shown to enhance the oxidative stability of packaged foods by regulating the release of bioactive compounds and inhibiting oxygen and moisture penetration (Li et al., 2015; Nath et al., 2022; Pires et al., 2018). Plant-derived EOs, which are designated as Generally Recognized as Safe (GRAS), are extensively used in the food sector due to their potent antioxidant and flavor-enhancing properties (Heydari et al., 2020). Myrtle (*Myrtus communis* L.), a perennial shrub belonging to the *Myrtaceae* family, is indigenous to the Mediterranean region, West Asia, and Iran. This medicinal plant is widely utilized across the food, cosmetic, perfumery, and medical industries due to its abundant bioactive compounds. Numerous studies have demonstrated that *M. communis* essential oil (MEO) exhibit strong antioxidant properties, primarily attributed to its high phenolic content (Barazandeh, 2000; Brahmi et al., 2023).

Several studies have evaluated the effects of plant-based mucilage coatings (Alizadeh Behbahani & Imani Fooladi, 2018; Behbahani et al., 2024; Majdi et al., 2024; Tanavar et al., 2021; Zanganeh et al., 2021) and other edible coatings (El Sheikha, Allam, ElObeid, et al., 2022) containing various biopreservatives on extending the shelf life of different types of meat. In addition, numerous researchers have examined the effects of edible films or coatings incorporating various EOs on the oxidative stability and/or sensory properties of ostrich meat (Ghaderi et al., 2024; Ghorbanlou et al., 2023; Heydari et al., 2020; Kiakojori et al., 2024). On the other hand, only one study has explored the antioxidant properties of directly adding MEO to minced beef (Dhifi et al., 2020). Currently, no known studies have focused on comparing the antioxidant effects of MEO when incorporated into conventional or nanocomposite food packaging systems with respect to the oxidative stability and sensory characteristics of muscle foods. Therefore, considering the nutritional benefits and oxidative susceptibility of ostrich meat, the desirable antioxidant properties of MEO, the appropriate film-forming properties of the *M. neglecta* leaf mucilage (MLM), and the necessity of developing active packaging systems using biodegradable nanocomposite coatings containing natural preservatives, this study aimed to: 1) Extract MEO and examine its chemical constituents; 2) Develop and characterize both conventional and nanocomposite (incorporating nanoclay) coatings made from MLM containing different concentrations of MEO; and 3) Compare the oxidative stability and sensory features of ostrich meat coated with different conventional and nanocomposite MLM-MEO coatings during 21-day storage at 4 °C.

## 2 Materials and methods

### 2.1 Materials

Fresh ostrich thigh meat was obtained directly from a local store in Zanjan City, Iran. *M. communis* (herbarium number: Tari 1350) and *M. neglecta* (herbarium number: Tari 2470) were purchased from the National Botanical Garden in Tehran, Iran. All chemicals and reagents including nanoclay montmorillonite powder, butylated hydroxytoluene (BHT), Folin–Ciocalteau reagent, sodium carbonate (Na_2_CO_3_), potassium iodide (KI), gallic acid, 1,1,3,3-tetraethoxypropane (TEP), sodium thiosulfate (Na_2_S_2_O_3_), calcium chloride (KCl), hydrochloric acid (HCl), 2-thiobarbituric acid (TBA), trichloroacetic acid (C_2_HCl_3_O_2_), perchloric acid (HClO_4_), anhydrous sodium sulfate (Na_2_SO_4_), 2,4-dinitrophenylhydrazine (DNPH), bovine serum albumin (BSA), acetic acid, starch solution, ethyl acetate, sodium phosphate buffer, guanidine hydrochloride, Tween 80, chloroform, methanol, and ethanol were supplied by Sigma Aldrich Company (Saint Louis, MO, USA).

### 2.2 *Myrtus communis* essential oil extraction

The *M. communis* leaves were thoroughly washed with tap water, left to dry at room temperature (25 ± 1 °C) under circulated air. They were ground into a powdered form using a mixer grinder to facilitate MEO extraction. Then, 100 g of the ground plant substance was placed in the Clevenger distillation flask (KOL, Behr, Düsseldorf, Germany), and distilled water was added to achieve a final volume of 1000 mL. The mixture was distilled at 100 °C for 45 min, repeating the process as needed to obtain the desired amounts of MEO. The MEO obtained was dehydrated using anhydrous Na_2_SO_4_ and stored in dark glass bottles at 4 °C until further use (Barazandeh, 2000).

### 2.3 Analysis of the chemical components of MEO

The Gas Chromatography-Mass Spectrometry (GC–MS) analysis was performed using a Hewlett Packard model 5890 Series II gas chromatograph with a flame ionization detector (FID) and incorporates both HP-WAX and DB-5 capillary columns, each was 30 m long, with an inner diameter of 0.25 mm and a film thickness of 0.25 μm. The temperature ranged from 50 °C to 265 °C with a gradient of 2.5 °C/min. The injector was kept at temperature of 250 °C, and the detector operated with an ionization energy set to 70 eV. Helium acted as the carrier gas, flowing at a rate of 1.2 mL/min, with a split ratio of 20:1. The chemical compounds present in MEO were determined by analyzing their retention values in comparison to published data and typical mass spectral fragmentation patterns (Wiley/NBS) as well as with the database of the National Institute of Standards and Technology (NIST) (Brahmi et al., 2023).

### 2.4 *Malva neglecta* leaf mucilage extraction

*M. neglecta* leaves were ground using a mixer grinder to a fine powder and defatted three times with 80 % ethanol for 2 h at 60 °C. The defatted powder (20 g) was then mixed with water at a 1:21 (*w*/*v*) ratio and heated for 4 h at 90 °C. The undissolved particles were separated using a nylon fabric, and the resulting filtrate was concentrated and treated with ethanol to induce precipitation. The extracted *Malva neglecta* leaf mucilage (MLM) was then dried at 50 °C, ground into a fine powder, packed, and preserved at 4 °C until further use (Sabahi et al., 2022).

### 2.5 Preparation of conventional and nanocomposite MLM coatings containing MEO

In this study, conventional MLM coatings were prepared in accordance with the method outlined by Sabahi et al. (2022), with some minor modifications. MLM powder (5 g) was combined with Tween 80 (1.75 g, equivalent to 35 % of the MLM powder) and dissolved in 100 mL of distilled water. During the stirring process, the mixture was heated to 80 °C for 30 min to obtain a uniform coating-forming solution (CFS). The CFS temperature was reduced to 25 °C. Based on preliminary test results (data not shown), different concentrations of MEO (0, 2, 4, and 8 % w/v) were individually added to the CFSs. The CFSs were then homogenized at 12,000 rpm for 5 min at 25 °C.

For the preparation of MLM nanocomposite coatings, the CFSs were initially prepared as described above. Nanoclay powder (3 % *w*/w of MLM) was added to the CFSs and mixed using a homogenizer at 5000 rpm for 10 min. After cooling, MEO and Tween 80 were added to the CFSs at concentrations equivalent to those used in the conventional coating. The CFSs were further homogenized at 20000 rpm for 5 min. To achieve nanoscale particle sizes, sonication was performed using an ultrasonic homogenizer (Qsonica, Q700, Newtown, CT, USA) with 700 W power, a frequency of 20 kHz, an amplitude of 50 %, and a pulse sequence of 5 s on, 5 s off, for 5 min at 25 °C. The sonication probe was immersed 20 mm below the CFS surface, and the containers were placed in an ice bath to prevent overheating (Mozafar et al., 2025).

### 2.6 Analysis of polydispersity index (PDI) and particle size of MLM nanocomposite coatings

The PDI and particle size of the MLM nanocomposite coatings were quantified by the dynamic light scattering (DLS) technique, employing a Zetasizer device (Malvern Panalytical Ltd., Malvern, UK) (Mozafar et al., 2025).

### 2.7 Application of conventional and nanocomposite MLM coatings containing MEO on ostrich meat

#### 2.7.1 Ostrich meat preparation

Fresh ostrich thigh meat was promptly transported to the laboratory in a polystyrene container with ice packs, under hygienic conditions. The meat was then washed with sterile water and sliced into 25 g portions, each approximately measuring 5 × 5 × 1 cm. The ostrich meat pieces were divided into nine groups. For each treatment, each group was immersed in a separate container containing a specific CFS (either conventional or nanocomposite, with a defined MEO concentration) for 3 min, followed by drying under a laminar hood at 25 °C for 10 min. An uncoated group was included as a control. The nine experimental groups evaluated in this study are summarized in Table 1. The samples were separately stored in sealed polyethylene bags at 4 °C and tested on days 0, 7, 14, and 21.

**Table 1:** Different experimental groups and their abbreviations.

No.	Treatment	Description
1	CON	Uncoated ostrich meat sample (Control)
2	MLM	Ostrich meat samples treated with conventional *Malva neglecta* leaf mucilage coating
3	MLM-MEO 2 %	Ostrich meat samples treated with conventional *Malva neglecta* leaf mucilage coating containing 2 % *Myrtus communis* essential oil
4	MLM-MEO 4 %	Ostrich meat samples treated with conventional *Malva neglecta* leaf mucilage coating containing 4 % *Myrtus communis* essential oil
5	MLM-MEO 8 %	Ostrich meat samples treated with conventional *Malva neglecta* leaf mucilage coating containing 8 % *Myrtus communis* essential oil
6	NMLM	Ostrich meat samples treated with nanocomposite *Malva neglecta* leaf mucilage/nanoclay coating
7	NMLM-MEO 2 %	Ostrich meat samples treated with nanocomposite *Malva neglecta* leaf mucilage/nanoclay coating containing 2 % *Myrtus communis* essential oil
8	NMLM-MEO 4 %	Ostrich meat samples treated with nanocomposite *Malva neglecta* leaf mucilage/nanoclay coating containing 4 % *Myrtus communis* essential oil
9	NMLM-MEO 8 %	Ostrich meat samples treated with nanocomposite *Malva neglecta* leaf mucilage/nanoclay coating containing 8 % *Myrtus communis* essential oil

#### 2.7.2 pH measurement

A pH meter (E520, Metrohm, Herisau, Switzerland) was first calibrated with two buffer solutions (pH 7 and pH 4), with temperature compensation set at 25 °C. The electrode was then rinsed with distilled water and wiped with a lint-free tissue. Following the manufacturer's instructions, 5 g of meat was homogenized in 25 mL of distilled water for about 30 s, and the pH was measured by submerging the electrode into each prepared sample (El Sheikha, Allam, Oz, et al., 2022).

#### 2.7.3 Total phenolic content

Total phenolic content (TPC) in ostrich meat samples was determined following the Liu et al. (2009)‘s method with minor modifications. Fifty g of ground ostrich meat was mixed with 92.5 mL of distilled water, boiled, and extracted for 20 min. Following cooling, the solution was filtered and combined with Folin–Ciocalteau (2.5 mL) reagent and a concentrated Na_2_CO_3_ (5 mL) solution. The resulting mixture was then stored in a dark environment for 1 h. The TPC was measured using a spectral photometer (DR 5000, HACH, Düsseldorf, Germany) at a wavelength of 700 nm. A calibration graph was established using phenolic acid, with data shown as mg GAE/g meat according to the following equation:

T = C·V/M.

Where *T* represents the TPC, *C* is the concentration of phenolic acid derived from the standard curve (mg/mL), *V* is the volume of solvent (mL), and *M* is the sample weight (g).

#### 2.7.4 Peroxide value

A 30 g sample of ostrich meat was mixed with 90 mL of a chloroform and methanol solvent (2:1, *v*/v) and homogenized for 5 min using a mixer. Following filtration, 30 mL of KCl (0.88 %, *w*/*v*) solution was added to the mixture. The obtained solution was transferred to a separating funnel and allowed to stand for 24 h to facilitate oil extraction. We extracted the lower phase and subjected to two additional extractions with 100 mL of a methanol/KCl (0.88 %, w/v) solution (1:1, *v*/v). Subsequently, the solution was removed using a rotary evaporation device at 35 °C. The peroxide value (PV) was measured by blending 1 g of the lipid extract with 30 mL of an acetic acid/chloroform solution (3:2, v/v). Following this, 0.5 mL of concentrated KI was combined with the solution, which was then incubated in a dark environment for 1 min. Subsequently, 30 mL of distilled water and 0.5 mL of a starch solution (1 % w/v) were incorporated into the sample. We conducted a titration with Na_2_S_2_O_3_ (0.01 N). The PV results were expressed as milliequivalents of peroxide oxygen per kilogram of lipid (meq/kg lipid) (Amiri et al., 2019).

#### 2.7.5 Thiobarbituric acid-reactive substances

The thiobarbituric acid reactive substances (TBARS) values were analyzed by mixing 10 g of ostrich meat with 1 mL of BHT solution (0.5 %, w/v in ethanol) and 35 mL of HClO_4_ (4 %, v/v), followed by then homogenization at 4000 rpm for 2 min. The mixture was passed through Whatman filter paper No. 1 with a final volume of 50 mL using HClO_4_ (4 %, v/v). A 5 mL aliquot of the filtrate was combined with 5 mL of TBA solution (0.02 M) and heated in a water bath at 100 °C for 1 h. After cooling to room temperature (25 ± 1 °C), the absorbance was recorded at 532 nm using a spectrophotometer. Malondialdehyde (MDA) concentrations were calculated using a standard graph prepared with TEP. The TBARS results were reported in mg MDA/kg meat (Hashemi et al., 2023).

#### 2.7.6 Total carbonyl content

To determine the total carbonyl content, 1 g of ostrich meat was homogenized with 10 mL of KCl (0.15 M) using an Ultra-Turrax blender for 1 min. Two 0.5 mL samples of the homogenate were combined with an equal volume of C_2_HCl_3_O_2_ (10 %), followed by centrifugation at 5000 rpm for 5 min to remove the upper liquid phase. A single pellet received treatment with 1 mL of HCl (2 M) containing DNPH (0.2 %) for carbonyl content analysis. In contrast, the second pellet was treated with 1 mL of HCl (2 M) for protein quantification. The samples were kept at 25 °C for 1 h, after which 1 mL of C_2_HCl_3_O_2_ (10 %) was added. The samples were again vortexed and centrifuged at 5000 rpm for 5 min. The pellets were washed three times with 1 mL of an ethyl acetate/ethanol mixture (1:1) to remove residual DNPH and solubilized in 1.5 mL of sodium phosphate buffer (20 M) containing guanidine hydrochloride (6 M, pH 6.5). The mixture was then centrifuged for 2 min at 5000 rpm. The absorbance of proteins and carbonyls was measured at 280 nm and 370 nm, respectively. Protein concentration was determined using a standard curve prepared with BSA in sodium phosphate buffer (20 mM) containing guanidine hydrochloride (6 M, pH 6.5). The total carbonyl content was calculated using the following formula:

Total carbonyl content = Abs_370nm_/21.0 mM^−1^ cm^−1^ × 1000.

Where 21.0 mM^−1^ cm^−1^ is the molar coefficient of carbonyls (Amiri et al., 2019).

#### 2.7.7 Sensory analysis

To assess the sensory attributes of the ostrich meat samples, a panel of 10 trained judges (five male and five female non-smokers from the student and staff population of Zanjan University of Medical Sciences) was selected based on their preliminary performance in the pre-test. Before the evaluation, introductory sessions were conducted to ensure that all panelists comprehended and agreed on the sensory attributes to be assessed. The ostrich meat was prepared using a microwave oven following the manufacturer's guidelines. Panel members assessed each sensory attribute using a 9-point hedonic scale (1: Extremely dislike, 9: Extremely like). Evaluations were conducted in private booths under incandescent lighting, and panelists rinsed their palates between assessments with unsalted crackers and water at 25 °C. Samples were randomly coded and assessed in a semi-blind manner. The fresh ostrich meat was used as a reference. Samples receiving scores above 4 were considered acceptable (Heydari et al., 2020). Taste characteristics were assessed only on days 0 and 7. Approval for the study was granted by the Ethics Committee of Zanjan University of Medical Sciences (Ethics Code: IR.ZUMS.BLC.1401.054), and informed consent was obtained from all individuals.

### 2.8 Statistical analysis

All measurements were repeated three times, and descriptive data were presented as Mean ± SE. All analyses were conducted using IBM SPSS Statistics (Version 26 for Windows, IBM Inc.), and all charts were generated using Microsoft Excel 2013. To measure the PDI and particle size of MLM nanocomposite coatings with different MEO concentrations, three independent batches were prepared for each type of coating solution and analyzed separately. The results were then compared using a one-way ANOVA and subsequently performing Tukey's honestly significant difference post hoc test (α = 0.05).

In the study on ostrich meat, nine experimental groups were established: (1) CON, (2) MLM, (3) MLM + MEO 2 %, (4) MLM + MEO 4 %, (5) MLM + MEO 8 %, (6) NMLM, (7) NMLM+MEO 2 %, (8) NMLM+MEO 4 %, (9) NMLM+MEO 8 %. Three independent batches were prepared for each group, and samples were randomly chosen at each of the four time points (0, 7, 14, and 21 days). All analyses (pH, TPC, PV, TBARS, and TCC) were performed separately for each sample. A General Linear Model was used for these tests, considering treatments and storage period as fixed factors, while their interaction was also examined. Tukey's post hoc test was applied to find significant differences (α = 0.05).

In the sensory evaluation, treatments and time intervals were treated as fixed variables, whereas the panelist group was considered as a random variable. The interaction between fixed and random factors was included in the model. Tukey's test was used to identify significant differences (α = 0.05).

## 3 Results and discussion

### 3.1 Chemical constituents of MEO

Table 2 displays the chemical composition of MEO along with their respective percentages. The GC–MS analysis detected twenty-five distinct chemical constituents, accounting for 99.82 % of the total EO content. The predominant constituents were α-pinene (48.72 %), eucalyptol (13.45 %), limonene (8.52 %), and linalool (7.28 %). Additionally, several other compounds, including linalyl acetate, geranyl acetate, α-terpinyl acetate, ρ-cymene, and α-terpineol, contributed between 1 and 4 % to the MEO composition, while the remaining compounds were present at concentrations below 1 %. These findings are consistent with the study conducted by Barazandeh (2000). However, Brahmi et al. (2023) identified different primary compounds in MEO, including α-pinene, 1.8-cineol, myrtenyl acetate, and d-limonene. Temperature, geographic location, nutrient availability, and photoperiod, significantly influence the chemical constituents of MEO by affecting the biosynthetic pathways in plants (Brahmi et al., 2023).

**Table 2:** Chemical constituents of *Myrtus communis* L. essential oil.

No.	Compound	Area (%)	RT⁎	RI⁎⁎	KI⁎⁎⁎
1	α-Thujene	0.61	5.15	932	931
2	α-Pinene	48.72	5.27	939	937
3	Sabinene	0.31	5.93	983	966
4	Myrcene	0.28	6.09	993	988
5	α-phellandrene	0.81	6.34	1010	1006
6	3-Carene	0.36	6.43	1016	1009
7	ρ-Cymene	2.01	6.65	1030	1021
8	Limonene	8.52	6.71	1034	1029
9	Eucalyptol	13.45	6.77	1038	1031
10	γ-Terpinene	0.69	7.17	1064	1057
11	Terpinolene	0.73	7.63	1094	1090
12	Linalool	7.28	7.76	1102	1094
13	α-Terpineol	1.62	9.18	1197	1189
14	Methyl chavicol	0.44	9.28	1204	1196
15	Linalyl acetate	3.87	10.04	1258	1256
16	Carvacrol	0.37	10.7	1305	1312
17	α-Terpinyl acetate	2.61	11.39	1357	1351
18	Neryl acetate	0.38	11.52	1366	1366
19	Geranyl acetate	3.12	11.78	1386	1379
20	Allylveratrole	0.56	12.08	1409	1404
21	Trans-Caryophyllene	0.91	12.43	1436	1418
22	λ-Gurjunene	0.24	12.68	1456	1474
23	Caryophyllene	0.83	12.86	1470	1583
24	Spathulenol	0.27	14.39	1596	1588
25	Globulol	0.83	14.48	1603	1661
Total		**99.82**			

⁎ Retention time.

⁎⁎ Retention indices.

⁎⁎⁎ Kovats indices.

### 3.2 Size of particles and PDI

The particle size of the nanocomposite was assessed due to its substantial impact on the functional properties of nanoparticles, such as storage stability, bioavailability, and physicochemical characteristics (Mozafar et al., 2025). Size of particles and PDI of NMLM coatings containing different concentrations of MEO are detailed in Table 3. The particle diameters ranged from 118.07 to 456.53 nm. As can be seen, the nanoparticle size decreased with increasing MEO concentration. This finding is consistent with the Polat Yemiş et al. (2022) results on nanoemulsion-based alginate coatings at different MEO concentrations. Several factors can influence the size of nanoparticles, including the EO type and concentration, detergent properties, preparation techniques, the presence of nanofillers (such as clay nanoparticles), and environmental conditions (e.g., metal ions and pH, base polarity, and solution viscosity). The binding of MEO inside the multilayer structure of clay nanoparticles appears to be the primary factor influencing particle size. Moreover, surfactant molecules can facilitate the incorporation of EO and promote dispersion, thereby reducing particle size. Furthermore, ultrasound treatment contributes to a decrease in both average size of droplets and PDI by generating surface waves and small bubbles that disrupt oil droplets (Mozafar et al., 2025). When the EO concentration in the biopolymer solution is low, two phenomena may occur: surfactant molecules at the EO droplet interface may repel biopolymer chains, and/or the biopolymer chains may exhibit a greater affinity for free surfactant molecules than for the EO droplets on the surface, leading to the formation of larger particles (Artiga-Artigas et al., 2017). The PDI serves as an indicator of the variability in particle size distribution, with lower values indicating greater particle uniformity and stability (Mozafar et al., 2025). In this study, the PDI of the nanocomposite samples ranged from 0.52 to 0.92. The PDI of NMLM coatings increased with higher MEO concentration increased (Table 3), corroborating the findings of Mozafar et al. (2025). This increase can be attributed to the water-resistant traits of EO, which encourage aggregation within the medium. As the concentration of EO rises, this aggregation intensifies, resulting in increased instability and greater variability in nanoparticle distribution within the polymer matrix (Mozafar et al., 2025).

**Table 3:** Polydispersity index (PDI) and particle size of nanocomposite MLM coatings with different concentrations of MEO (Mean ± S.E.).

Sample	Concentration (%)	PDI	Particle size (nm)
NMLM	–	0.83 ± 0.11 ^a^	456.53 ± 26.16 ^a^
NMLM-MEO	2	0.52 ± 0.06 ^a^	162.93 ± 1.36 ^b^
	4	0.58 ± 0.08 ^a^	166.17 ± 13.79 ^b^
	8	0.92 ± 0.19 ^a^	118.07 ± 22.54 ^b^

NMLM: Nanocomposite *Malva neglecta* leaf mucilage coating solution; MLM-MEO: Nanocomposite *Malva neglecta* leaf mucilage coating solution containing different concentrations of *Myrtus communis* essential oil.

Values marked by different letters within the same column are significantly different according to Tukey's test (*P* ≤ 0.05).

### 3.3 pH

**Fig. 1:**
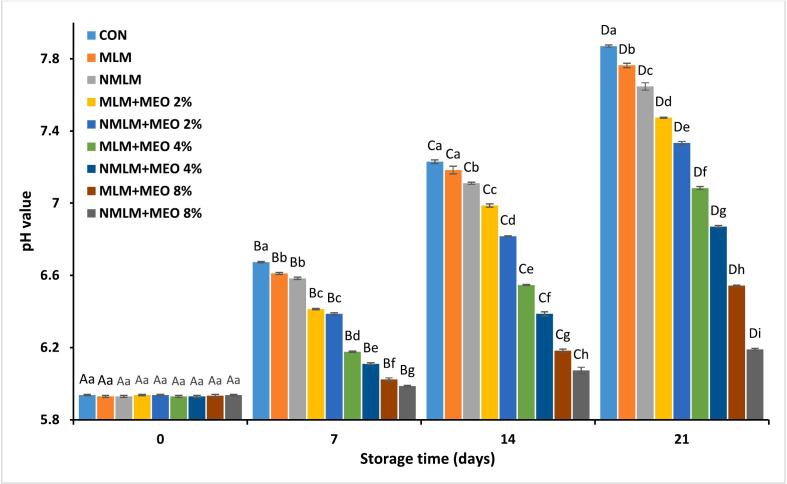
Changes in pH values of ostrich meat treated with conventional and nanocomposite MLM coatings containing different concentrations of MEO during 21 days at 4 °C (Mean ± S.E.). CON (control): Uncoated ostrich meat; MLM: Ostrich meat treated with conventional *Malva neglecta* leaf mucilage coating; NMLM: Ostrich meat sample treated with nanocomposite *Malva neglecta* leaf mucilage coating; MLM-MEO: Ostrich meat sample treated with conventional *Malva neglecta* leaf mucilage coating containing *Myrtus communis* essential oil; NMLM-MEO: Ostrich meat sample treated with nanocomposite *Malva neglecta* leaf mucilage coating containing *Myrtus communis* essential oil. Values marked by different capital letters within the same experimental group, as well as values marked with different lowercase letters within the same day, are significantly different according to Tukey's test (*P* ≤ 0.05).

pH fluctuations over time can serve as a marker for meat quality (Hashemi et al., 2023). Fig. 1 illustrates the pH of ostrich meat treated with conventional and nanocomposite MLM coatings across 21 days of storage at 4 °C. The initial pH values ranged from 5.93 to 5.94, consistent with prior studies (Amiri et al., 2019; Heydari et al., 2020). Over time, significant upward trends in pH values were observed across all experimental groups during the storage period (*P* ≤ 0.05). This increase can be attributed to microbial degradation of the ostrich meat tissue, resulting in protein breakdown and nitrogenous compounds release (Heydari et al., 2020). However, the increase in pH values in samples treated with MLM-MEO and NMLM-MEO coatings occurred at a slow rate compared to other groups. This can be explained by to the preventive effects of MEO phenolic compounds on bacterial proliferation, which reduces the degradation of amino compounds during storage. Fayazi et al. (2021) similarly observed a slower pH increase in turkey meat samples packaged with carboxymethylcellulose film containing MEO, attributing this to the antimicrobial effects of the EO. Additionally, samples treated with nanocomposite coatings exhibited lower pH values than those with conventional coatings at similar MEO concentrations. The most significant decrease in pH was observed in the NMLM-MEO 8 % treatment by the end of the storage period among all experimental groups (*P* ≤ 0.05). This can be explained by the enhanced antimicrobial properties of MEO at the nanoscale level when incorporated into the coating (Amiri et al., 2019). In support of these results, Ghaderi et al. (2024) found a slower increase in pH values of ostrich meat hamburgers treated with gelatin/chitosan nanocomposite film enriched with *Laurus nobilis* EO nanoemulsions compared to the other experimental groups.

### 3.4 Total phenolic content

**Fig. 2:**
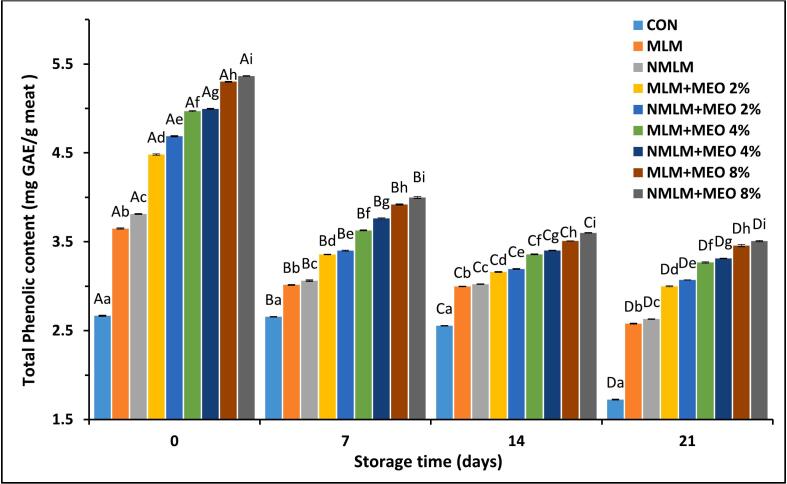
Changes in total phenolic content (TPC) in ostrich meat treated with conventional and nanocomposite MLM coatings containing different concentrations of MEO during 21 days at 4 °C (Mean ± S.E.). CON (control): Uncoated ostrich meat; MLM: Ostrich meat treated with conventional *Malva neglecta* leaf mucilage coating; NMLM: Ostrich meat sample treated with nanocomposite *Malva neglecta* leaf mucilage coating; MLM-MEO: Ostrich meat sample treated with conventional *Malva neglecta* leaf mucilage coating containing *Myrtus communis* essential oil; NMLM-MEO: Ostrich meat sample treated with nanocomposite *Malva neglecta* leaf mucilage coating containing *Myrtus communis* essential oil. Values marked by different capital letters within the same experimental group, as well as values marked with different lowercase letters within the same day, are significantly different according to Tukey's test (*P* ≤ 0.05).

Phenolic compounds in meat play a crucial role in protecting against oxidation processes (Saleh et al., 2017). Fig. 2 presents the TPC in ostrich meat treated with conventional and nanocomposite MLM coatings containing different concentrations of MEO during 21 days of storage at 4 °C. Initial TPCs ranged from 2.67 to 5.36 mg GAE/g meat. The presence of phenolic compounds in the CON group indicated that extractable polyphenolic compounds in poultry feed can be taken up through the gastrointestinal tract and accumulate in the muscle (Saleh et al., 2017). Furthermore, the presence of TPC in MLM and NMLM treatments suggests that although plant-based mucilage primarily consists of polymeric polysaccharides, it may also contain small quantities of phenolic compounds, which is consistent with other studies (Kučka et al., 2024). The TPC of ostrich meat samples treated with MLM-MEO and NMLM-MEO was significantly higher than that of the MLM and NMLM treatments, likely due to the phenolic content in MEO (Yaghoubi et al., 2021). During the storage period, there was a notable decrease in phenolic compounds across all meat samples (*P* ≤ 0.05), which aligns with the findings of other studies (Liu et al., 2009; Yaghoubi et al., 2021). The observed reduction in phenolic components is attributable to oxidation reactions occurring during storage (Yaghoubi et al., 2021). By the end of the storage period, the NMLM-MEO 8 % exhibited the highest TPC among all experimental groups (*P* ≤ 0.05). This may be due to the presence of nanoclay in the nanocomposite coating, which facilitates controlled release of phenolic compounds and prevents their evaporation (Nath et al., 2022).

### 3.5 Lipid oxidation

#### 3.5.1 Peroxide value

**Fig. 3:**
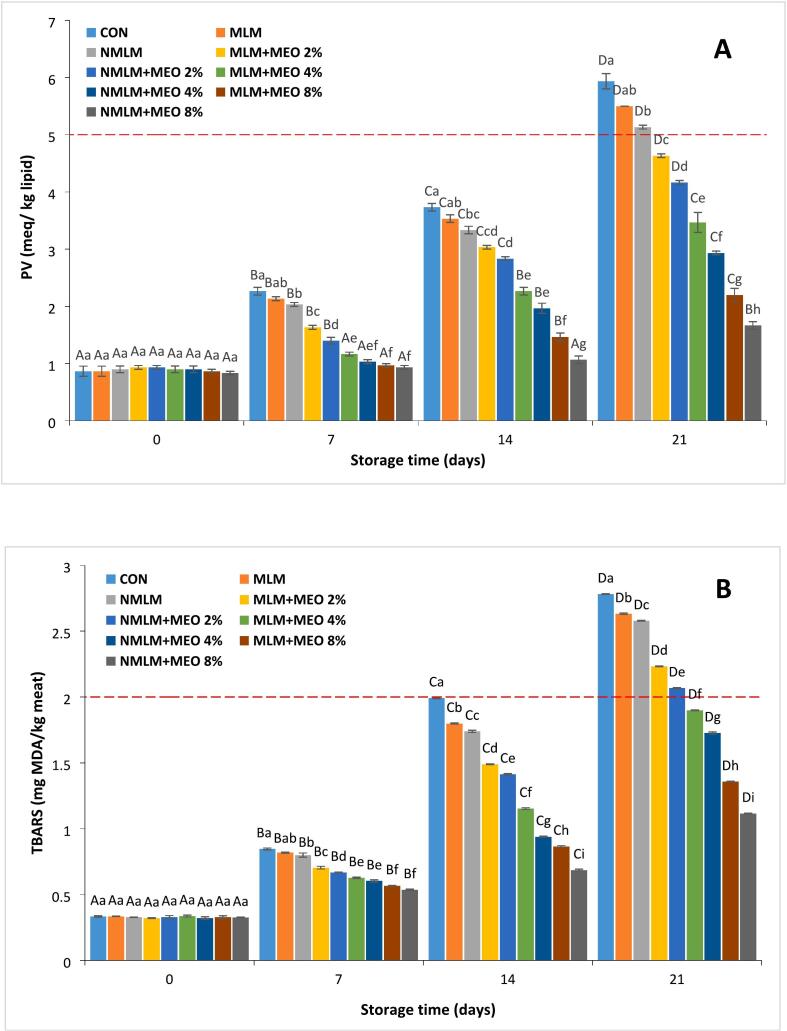
Changes in (A) Peroxide value (PV) and (B) Thiobarbituric acid-reactive substances (TBARS) in ostrich meat treated with conventional and nanocomposite MLM coatings containing different concentrations of MEO during 21 days at 4 °C (Mean ± S.E.). CON (control): Uncoated ostrich meat; MLM: Ostrich meat treated with conventional *Malva neglecta* leaf mucilage coating; NMLM: Ostrich meat sample treated with nanocomposite *Malva neglecta* leaf mucilage coating; MLM-MEO: Ostrich meat sample treated with conventional *Malva neglecta* leaf mucilage coating containing *Myrtus communis* essential oil; NMLM-MEO: Ostrich meat sample treated with nanocomposite *Malva neglecta* leaf mucilage coating containing *Myrtus communis* essential oil. Values marked by different capital letters within the same experimental group, as well as values marked with different lowercase letters within the same day, are significantly different according to Tukey's test (*P* ≤ 0.05).

The peroxide value (PV) is an indicator of lipid oxidation, measuring levels of peroxides and hydroperoxides, which are the initial oxidation products (Najafi et al., 2022). Fig. 3A demonstrates the PV of ostrich meat treated with conventional and nanocomposite MLM coatings containing various concentrations of MEO over 21 days of storage at 4 °C. Initial PVs of all experimental groups ranged from 0.87 to 0.93 meq/kg of lipid, consistent with previous studies (Abou-Arab & Abu-Salem, 2010; Najar et al., 2024). Consequently, the PVs increased significantly by the end of the storage period (*P* ≤ 0.05). According to Najar et al. (2024), the maximum permissible PV for ostrich meat is 5 meq/kg of lipid. The PVs of MLM-MEO and NMLM-MEO treatments remained significantly lower than those of the MLM and NMLM treatments, staying below the permissible limit (*P* ≤ 0.05). Earlier research has demonstrated that phenolic compounds can inhibit lipid free radical formation and interact with oxygen during the oxidation process, thereby delaying lipid oxidation onset (Boroujeni & Hojjatoleslamy, 2018). These results corroborate findings from Boroujeni and Hojjatoleslamy (2018), who investigated the use of MEO to reduce PV in frying oil used for potato chips. Furthermore, PVs of NMLM-MEO samples were lower than those of MLM-MEO treatments at equivalent MEO concentrations. The greatest decrease in PV was observed in the NMLM-MEO 8 % treatment (*P* ≤ 0.05). This effect may be explained by the enhanced oxidative stability associated with the reduction in nanoparticle size, which delays the initial oxidation process and production of hydroperoxides (Amiri et al., 2019). Similarly, Ghaderi et al. (2024) reported improvements in PV for ostrich meat hamburgers treated with gelatin/chitosan nanocomposites enriched with *L. nobilis* EO nanoemulsions. Li et al. (2015) suggested that delay in PV increases in fresh pork meat treated with gelatin films containing nanoclay, compared to conventional films. This occurs because of forming systematically arranged nanoclay layers in gelatin that create a complex way that decreases the permeability to oxygen and moisture. As a result, nanoclay-containing films exhibited significant antioxidant activities, substantially enhancing meat shelf life during storage (Li et al., 2015).

#### 3.5.2 TBARS value

The TBARS value is a commonly used indicator of rancidity, reflecting the collection of secondary products resulting from lipid oxidative reactions in meat products (Hashemi et al., 2023). Fig. 3B illustrates the TBARS values of ostrich meat treated with conventional and nanocomposite MLM coatings containing various concentrations of MEO during 21 days of storage at 4 °C. Initial TBARS values ranged from 0.32 to 0.34 mg MDA/kg meat, similar to values stated in previous studies (Abou-Arab & Abu-Salem, 2010). Throughout the storage period, TBARS values for all treatments showed a significant upward trend, peaking on day 21, with values ranging from 1.12 to 2.78 mg MDA/kg meat (*P* ≤ 0.05). Treatments with varying MEO concentrations demonstrated lower TBARS values than the other groups (*P* ≤ 0.05), likely due to the antioxidant properties of phenolic compounds in MEO, consistent with previous studies (Boroujeni & Hojjatoleslamy, 2018; Dhifi et al., 2020). Hoffman et al. (2014) established a maximum permissible TBARS value of 2 mg MDA/kg sample for an ostrich-based meat product, beyond which off-flavors were detectable in sensory evaluation. Findings from the present study indicated that the TBARS index remained below this threshold in all MLM-MEO and NMLM-MEO treatments containing 4 % and 8 % EO at the end of the storage period. Furthermore, NMLM-MEO samples displayed lower TBARS levels than MLM-MEO treatments at equivalent MEO concentrations. The NMLM-MEO 8 % treatment achieved the most significant reduction in TBARS level when the storage duration finished (*P* ≤ 0.05), likely due to the controlled release of active antioxidant compounds from the nanocomposite coatings throughout storage (Ghaderi et al., 2024). In a study by Pires et al. (2018), chitosan/nanoclay bionanocomposites containing rosemary and ginger EOs demonstrated a delayed upward trend in TBARS values. This effect was attributed to the additional barrier provided by clay nanoparticles, which protect against ultraviolet rays and limit oxygen exchange between the food and its external environment.

### 3.6 Protein oxidation

**Fig. 4:**
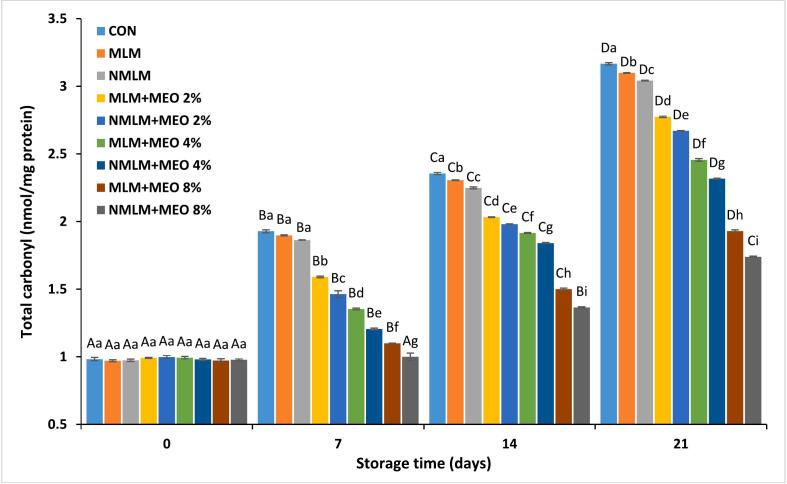
Changes in total carbonyl content in ostrich meat treated with conventional and nanocomposite MLM coatings containing different concentrations of MEO during 21 days at 4 °C (Mean ± S.E.). CON (control): Uncoated ostrich meat; MLM: Ostrich meat treated with conventional *Malva neglecta* leaf mucilage coating; NMLM: Ostrich meat sample treated with nanocomposite *Malva neglecta* leaf mucilage coating; MLM-MEO: Ostrich meat sample treated with conventional *Malva neglecta* leaf mucilage coating containing *Myrtus communis* essential oil; NMLM-MEO: Ostrich meat sample treated with nanocomposite *Malva neglecta* leaf mucilage coating containing *Myrtus communis* essential oil. Values marked by different capital letters within the same experimental group, as well as values marked with different lowercase letters within the same day, are significantly different according to Tukey's test (*P* ≤ 0.05).

Protein oxidation in muscle foods, which are rich in protein, reduces nutritional value and product quality, representing a significant yet often overlooked form of chemical deterioration. The total carbonyl content serves as a key indicator of protein oxidation (Domínguez et al., 2021). Fig. 4 illustrates the total carbonyl content of ostrich meat treated with conventional and nanocomposite MLM coatings containing various concentrations of MEO during a 21 days of storage at 4 °C. Initial total carbonyl content across all experimental groups varied between 0.97 and 0.99 nmol/mg protein. This value exhibited an upward trend during storage, peaking at 1.74 to 3.17 nmol/mg protein by day 21 (*P* ≤ 0.05). The synthesis of carbonyls occurs due to the chemical assault of reactive oxygen species (ROS) on alkaline amino acids inside muscle proteins, targeting the peptide backbone and the side chains of both aliphatic and aromatic amino acids. Furthermore, secondary lipid oxidation products, such as reactive aldehydes, can indirectly trigger protein oxidation (Domínguez et al., 2021). Previous studies have reported the relationship between protein and lipid oxidation in ostrich meat (Hoffman et al., 2014). Moreover, these findings can be attributed to the elevated iron concentration in ostrich meat, as this metal is known to be one of the most effective inducers of protein carbonylation in muscle foods (Domínguez et al., 2021). The carbonyl content in treatments with varying MEO concentrations was lower than in samples without MEO during storage (*P* ≤ 0.05), highlighting MEO's effectiveness as an organic preservative in preventing protein degradation. Phenolic substances containing free hydroxyl groups inhibit the oxidation of protein thiol groups, leading to a higher sulfhydryl content in treated meat compared to untreated groups (Amiri et al., 2019). Hoffman et al. (2014) attributed the oxidative stability of ostrich meat proteins to phenolic compounds present rooibos tea extract. In addition, NMLM-MEO samples displayed lower total carbonyl content than MLM-MEO treatments at equivalent MEO concentrations. The NMLM-MEO 8 % showed the lowest total carbonyl content among all experimental groups at the end of the storage period (*P* ≤ 0.05). These outcomes are consistent with studies investigating the impact of nanotechnology on the antioxidant features of edible coatings and films containing EOs to prevent protein oxidation in various muscle foods (Amiri et al., 2019; Ojeda-Piedra et al., 2022).

### 3.7 Sensory analysis

**Fig. 5:**
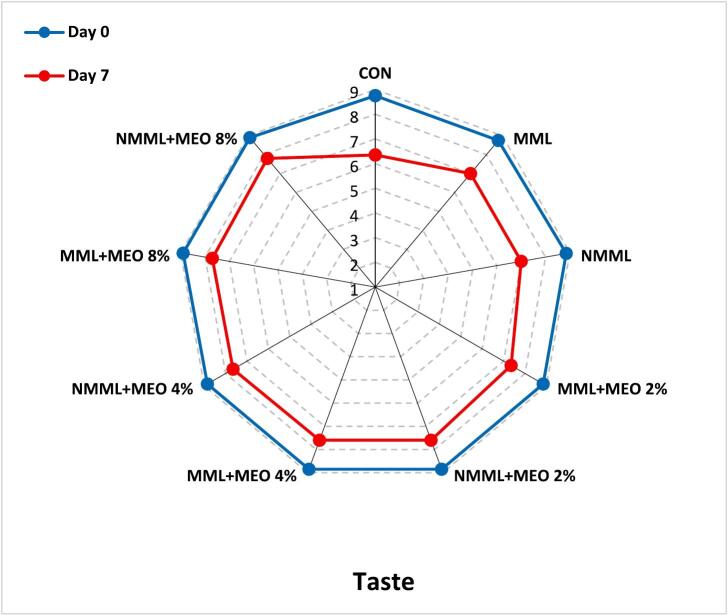

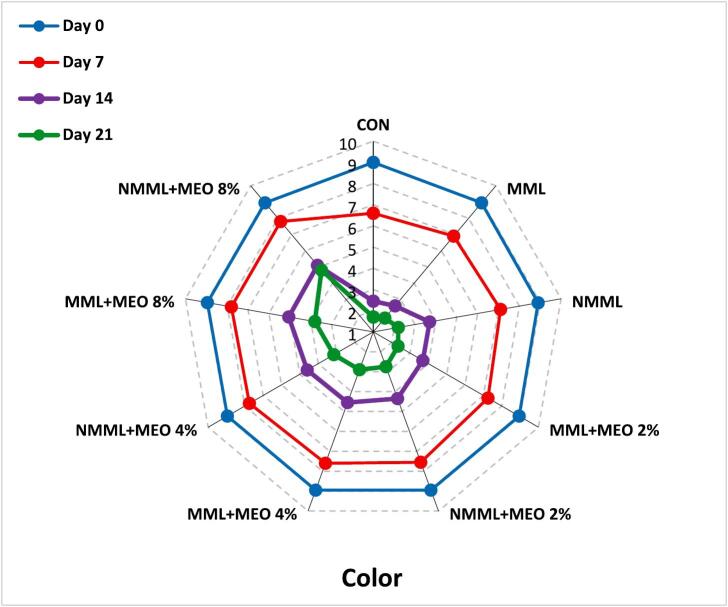

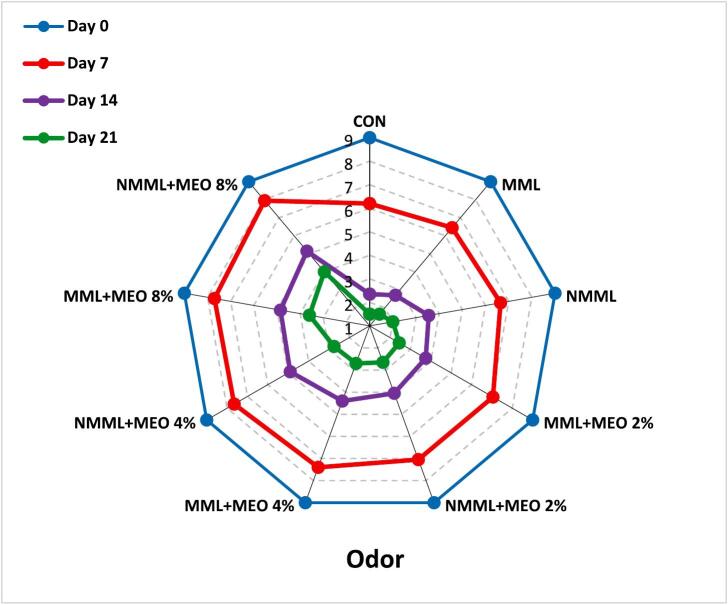

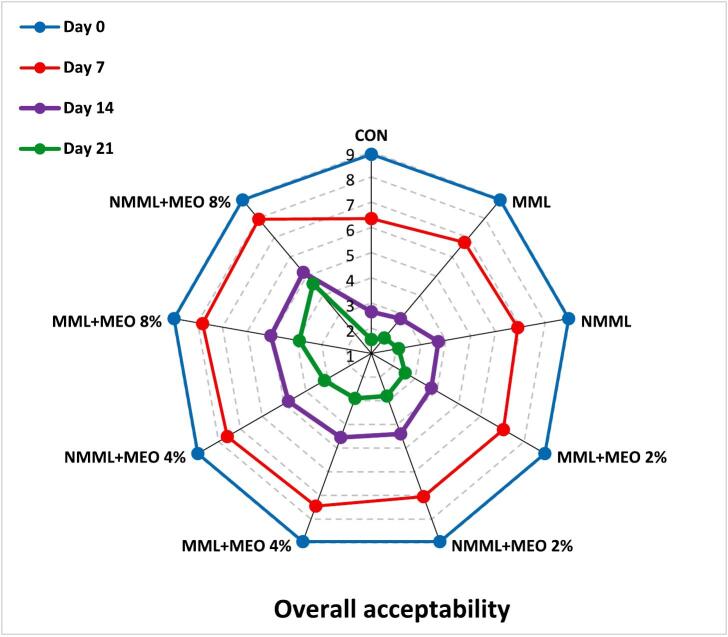
Changes in sensory characteristics of ostrich meat treated with conventional and nanocomposite MLM coatings with different concentrations of MEO during 21 days at 4 °C. CON (control): Uncoated ostrich meat; MLM: Ostrich meat treated with conventional *Malva neglecta* leaf mucilage coating; NMLM: Ostrich meat sample treated with nanocomposite *Malva neglecta* leaf mucilage coating; MLM-MEO: Ostrich meat sample treated with conventional *Malva neglecta* leaf mucilage coating containing *Myrtus communis* essential oil; NMLM-MEO: Ostrich meat sample treated with nanocomposite *Malva neglecta* leaf mucilage coating containing *Myrtus communis* essential oil.

Meat products are notably sensitive to sensory spoilage due to their complex structure, primarily consisting of water, proteins, and lipids. Therefore, sensory attributes are crucial for evaluating consumer acceptability (Hashemi et al., 2023). Fig. 5 illustrates the changes in sensory characteristics of ostrich meat treated with conventional and nanocomposite MLM coatings containing various concentrations of MEO during a 21 days of storage at 4 °C. In the early stages of the storage, all treatment groups achieved acceptable scores across all sensory characteristics. However, sensory scores showed a declining trend throughout the storage period (*P* ≤ 0.05). Due to relatively unpleasant odors, the taste attribute was not evaluated after day seven. The results indicated that the CON, MLM, and NMLM treatments had lower acceptability scores during the storage period compared to other groups (*P* ≤ 0.05). By day 14, sensory scores for these treatments fell below the unacceptable threshold of 4, likely due to spoilage signs such as discoloration and off-flavor. Supporting these results, Dhifi et al. (2020) reported that different concentrations of MEO improved sensory properties in ground beef during refrigerated storage. These results indicate a direct link between lower sensory scores and the oxidation of proteins and lipids. Byproducts of oxidation, including esters, ketones, aldehydes, hydrocarbons, ammonia, and alcohols, as well as microbial activity, play a role in meat spoilage, which is characterized by discoloration, slime formation, off-odors, and off-flavors (Amiri et al., 2019). Additionally, NMLM-MEO treatments demonstrated superior sensory scores compared to MLM-MEO treatments at the same EO concentrations. The NMLM-MEO 8 % treatment achieved the highest sensory scores among all groups (*P* ≤ 0.05) and was the only treatment to exceed the acceptable limit by the end of the storage period. Ghaderi et al. (2024) similarly confirmed the correlation between increased TBARS levels and reduced sensory properties of ostrich meat-based hamburgers treated with gelatin/chitosan nanocomposite films containing L. *nobilis* EO nanoemulsions. Additionally, a study comparing the impacts of chitosan coatings integrating free or nanoencapsulated Satureja EO on the quality of lamb meat found that coatings enriched with nanoencapsulated EO had the greatest impact on improving sensory properties (Pabast et al., 2018).

## 4 Conclusion

This research suggests that the incorporation of MEO into both conventional and nanocomposite MLM coatings significantly enhanced the total phenolic content and, consequently, the antioxidant activity of ostrich meat stored for 21 days at 4 °C. Notably, the nanocomposite MLM coatings exhibited higher antioxidant activity than the conventional MLM coatings at the same MEO concentrations. Among the treatments, samples with the nanocomposite MLM coatings containing 8 % MEO (NMLM-MEO 8 %) showed the best oxidative stability and sensory properties. This particular treatment extended the shelf life of ostrich meat by at least 7 days, based on established chemical and sensory acceptability thresholds. From an application perspective, these findings highlight the potential of the NMLM-MEO 8 % coating as an effective active packaging system to prevent oxidative deterioration in muscle foods, offering a sustainable and natural alternative for the meat packaging industry. However, despite the nanoclay content in the NMLM coating being considered safe at the studied levels, comprehensive toxicological evaluations and migration studies under real packaging conditions are recommended. Furthermore, given MEO's recognized antimicrobial properties, further research is required to investigate and compare the antimicrobial effects of MLM-MEO and NMLM-MEO coatings. Exploring the performance of NMLM-MEO nanocomposites on different meat types and storage conditions, as well as evaluating their scalability for industrial applications, are also promising directions for future research.

## CRediT authorship contribution statement

**Atefeh Karimi:** Writing – original draft, Visualization, Validation, Software, Methodology, Investigation, Formal analysis, Data curation. **Majid Aminzare:** Writing – review & editing, Visualization, Validation, Supervision, Software, Resources, Project administration, Methodology, Investigation, Funding acquisition, Formal analysis, Data curation, Conceptualization. **Hassan Hassanzadazar:** Writing – review & editing, Validation, Supervision, Resources, Project administration, Methodology, Funding acquisition, Conceptualization. **Mahsa Hashemi:** Writing – review & editing, Visualization, Validation, Supervision, Investigation, Formal analysis, Data curation. **Shahin Roohinejad:** Writing – review & editing, Validation, Project administration, Methodology, Formal analysis, Conceptualization. **Alaa El-Din Ahmed Bekhit:** Writing – review & editing, Validation, Project administration, Methodology, Investigation, Formal analysis, Conceptualization. **Reza Tahergorabi:** Writing – review & editing, Validation, Software, Methodology, Investigation, Conceptualization.

## Declaration of competing interest

The authors declare that they have no known competing financial interests or personal relationships that could have appeared to influence the work reported in this paper.

## Data Availability

Data will be made available on request.
